# Thermorheological Characterization of Healthier Reduced-Fat Cocoa Butter Formulated by Substitution with a Hydroxypropyl Methylcellulose (HPMC)-Based Oleogel

**DOI:** 10.3390/foods10040793

**Published:** 2021-04-07

**Authors:** María Dolores Alvarez, Susana Cofrades, María Espert, Ana Salvador, Teresa Sanz

**Affiliations:** 1 Instituto de Ciencia y Tecnología de Alimentos y Nutrición (ICTAN-CSIC), 28040 Madrid, Spain; scofrades@ictan.csic.es; 2 Instituto de Agroquímica y Tecnología de Alimentos (IATA-CSIC), 46980 Paterna, Spain; mespert@iata.csic.es (M.E.); asalvador@iata.csic.es (A.S.); tesanz@iata.csic.es (T.S.)

**Keywords:** cocoa butter, hydroxypropyl methylcellulose, emulsion-templated approach, edible oleogel, thermorheological properties, fatty acids profile

## Abstract

Cocoa butter (CB) is a main ingredient in pastry due to the unique functional properties of its fat, which is high in saturated fatty acids (SFAs). However, excessive consumption of SFAs is associated with the occurrence of several chronic diseases. This study researched the partial or total replacement of CB by an oleogel (OG) formulated with a healthier lipid profile, for mixed systems that would allow a partial substitution of CB in confectionery products. The “emulsion-templated approach” was used to develop a sunflower oil-HPMC-based OG. Different CB:OG ratios were formulated increasing the percentage replacement of CB by OG from 50 to 100%. Rheological and textural properties were determined and compared with a CB control at 20 and 10 °C. Oil-binding capacity was also analyzed. The systems showed a solid-like behavior, with higher elastic than viscous modulus, which increased with CB concentration. Compared with 20 °C, at 10 °C there was an increase in connectivity, viscoelasticity, and consistency of the systems, in response to a more complete CB crystallization. The replaced systems also presented a better lipid profile than CB. This evidence suggests that formulated CB:OG system at 50:50 ratio could become useful as a CB equivalent in chocolate products.

## 1. Introduction

Recently, consumer awareness of the relationship between diet and health has increased reduction of saturated fatty acids SFAs and elimination of trans-fatty acids (FAs) present in solid fats by substituting them with essential and polyunsaturated fatty acids (PUFAs), to diminish the risk of cardiovascular disease, obesity and diabetes [1,2,3], which converges with the World Health Organization (WHO) recommendations [4]. However, saturated fats are responsible for many high-quality attributes in foodstuffs, such as aroma, stability, taste and texture, and therefore, both fat reduction and fat replacement are not easy tasks for food manufacturers [5,6]. Substituting saturated fats (solid at room temperature) with liquid oils alters the behavior of fat and of products made with fat replacers, especially fat crystallization and specific melting profile, which affect sensory attributes such as spreadability, mouthfeel, snap of chocolate, hardness, palatability, etc. [6,7].

Currently, manufacturers and researchers are focusing on different technological options to structure liquid oils for their use as saturated fat replacers, hence mimicking fat functionality in food products without changing the nutritional profile of liquid oils [1,8]. In this context, organogelification converts liquid oils into soft matter structures with solid-lipid functionality in line with that of saturated fats [1,2,9]. Oleogels are structured oils prepared by oleogelation of liquid oils using oleogelators, which have features (rheological properties, viscoelasticity, plasticity, consistency, etc.) of solid fats, but contain a low quantity of SFAs [10,11]. Oleogelators can be classified as polymeric and low-molecular weight [2,12]. Among them, current research focus is the use of hydrocolloids as oil structuring agents through indirect methods [13,14,15,16,17].

The emulsion-templated approach for oil structuring is described as an indirect method to obtain oleogels by first creating a water continuous emulsion, followed by removing water and further shearing the dried emulsion [18,19,20]. These oleogels can be considered to be fat analogs as they contain a high concentration (above 97 wt.%) of edible oil [11]. However, oil droplets in concentrated emulsions tend to coalesce during the drying process [17]. To avoid coalescence of oil droplets, the creation of a strengthened emulsion interface by using thickener agents has shown to be effective in combinations such as regenerated cellulose and carboxymethyl cellulose [21] and methylcellulose/hydroxypropyl methylcellulose and xanthan gum [13,14,15,16]. Therefore, the research of single or combined hydrocolloids as oil-gelling agents and new sustainable indirect methods as the emulsion-templated approach, would greatly contribute to increasing the field of application of oleogels and the generalization of their use in food technology.

Regarding the production of oleogels, there is limited literature concerning the use of hydroxypropyl methylcellulose (HPMC) [14]. The authors just cited used the emulsion-templated method to prepare oleogels with soybean oil and a wide range of HPMC concentrations, observing that the mechanical strength increased with HPMC concentration. Recently, Espert et al. [18] have pointed out the suitability of HPMC emulsions without additional thickeners to obtain oleogels. HPMC use has also proven to be a good strategy to diminish the effective caloric content of foods, and to control fat digestion or satiety [5,22,23].

Genuine cocoa butter (CB) is mainly used in pastry and confectionery to make both dark and white chocolate products due to its unique triacylglycerol (TAG) composition that provides the exclusive physical and sensory characteristics of this ingredient [24,25]. In turn, CB is the most expensive ingredient among chocolate products and, to minimize costs, cocoa butter equivalents, substitutes or improvers are typically used as CB replacers [24,26,27]. These alternative fat and oil sources are obtained by using chemical or enzymatic methods, to resemble the TAG of CB [28,29]. However, although CB equivalents are used to develop low-priced and appropriate alternatives to CB [25], they do not meet actual consumer demand for low fat foodstuffs with a healthier lipid profile, while retaining their technological and sensory characteristics.

The suitability of a HPMC-based OG obtained by the emulsion-templated approach for partial substitution of CB in confectionery products, improving their nutritional quality by reducing saturated fat content, has not been investigated. In this study, the objective was to investigate the effect of percentage replacement of CB by an OG enhanced with a healthy oil stabilized by HPMC on the thermorheological behavior and the consistency of the different CB:OG systems, using a combination of small amplitude oscillation sweeps (SAOS) and large deformation penetration texture tests. In addition, the oil-binding capacity (OBC) and the fatty acid (FA) composition of the CB:OG systems were also analyzed. It is expected that the obtained OG may partially substitute CB in chocolate products reducing their cost, but with the advantage of reducing intake of SFAs and that is compatible with the particular physical and sensory properties provided by CB.

## 2. Materials and Methods

### 2.1. Materials

The emulsion and the OG were prepared with high oleic refined sunflower oil (HORSO), drinking water, and a type of HPMC. Sunflower oil “Capicua” was purchased from Coreysa (Sevilla, Spain). HPMC (METHOCEL™ F4M Food Grade) was employed as organogelator and provided by The Dow Chemical Company (Bomlitz, Germany). This HPMC has chemical substitution of 29 g/100 g methoxyl and 6.8 g/100 g hydroxypropyl, its viscosity is of 4000 mPa s at 2% aqueous solution and 20 °C, its average molecular weight (*M*_n_) is of 86,000 as measured by The Dow Chemical Company. In addition, the polydispersity of this linear polymer is approximately 3.0. Barry white cacao pearls were employed in the preparation of the CB:OG systems as the CB source, and were supplied by Barry Callebaut Manufacturing Iberica S.A.U. (Barcelona, Spain).

### 2.2. Emulsion and Oleogel (OG) Preparation 

The oil-in-water emulsion stabilized by HPMC was composed of sunflower oil (47% *w/w*, 94 g), HPMC (1% *w/w*, 2 g) and drinking water (52% *w/w*, 104 g) for a total final mass of 200 g. The proportions were chosen in accordance with the results of a previous study where two oil concentrations (47 and 60%) and three HPMC concentrations (0.5, 1 and 2%) were compared [18]. HPMC was first dispersed in the oil using a Bunsen (AGV-8) rod stirrer at low speed (300 rpm) for 5 min. Then, the mixture was hydrated by gradually adding the water at 10 °C for 30 s. The 200 g mixture was then homogenized using a high-energy dispersing unit IKA T25 basic (Ultra-Turrax) with the dispersion tool S18N-19G, at 6500 rpm for 15 s, and subsequently at 17,500 rpm for 60 s. The resulting emulsion was then immediately poured into plastic trays (155 mm length × 140 mm width × 30 mm depth) forming a thin layer. 

The drying of the emulsion was carried out using forced convection air in a climate chamber (KBF240, Binder Iberica, Barcelona, Spain) at 60 °C for 30 h, obtaining sheets of about 10 mm thickness. The complete removal of water to 0.0–0.5% moisture content was confirmed by the weight difference between the emulsion and dried product. The total elimination of water from the emulsion resulted in the development of solid-like structures formed by HPMC, with a high proportion of embedded oil.

Following, the OG was prepared by uniformly shearing the dried product in accordance with Patel et al. [11] and Espert et al. [18], using a conventional A320R1 mincer (Moulinex, Gruope Seb Ibérica, Barcelona, Spain) and performing 5 cycles of 3 s of duration each. Then, the OG smooth mixture obtained was molded into plastic buckets (~12 g of product at each bucket (20 × 30 mm)) to attain a homogeneous oil-sorbed material, and finally cooled and stored at refrigeration temperature (4 °C) for 24 h until further analysis.

### 2.3. CB:OG Systems Preparation 

Five different CB:OG systems were prepared by partially (50, 60, 70 and 80%) and totally (100%) replacing CB by the OG (CB:OG, 50:50, 40:60, 30:70, 20:80 and 0:100), and a genuine CB system (100:0) without added OG was also formulated and used as control. Therefore, the effect of the replacement percentage was studied at a fixed total concentration. To prepare the CB:OG mixed systems, first, the cacao butter pearls were melted by placing them in a thermostatic water bath (Series BD, Bunsen, Madrid, Spain) at 75 °C to melt all the fat crystals, followed by the immediate cooling of the liquid butter to 60 °C. In turn, the corresponding quantity of OG required for each case was slightly heated to 40 °C by placing it in another thermostatic water bath (Univeba, JP Selecta, Barcelona, Spain) at this temperature under slight stirring at 300 rpm. Following, the melted CB was slowly added to the OG, also under continuous stirring. The mixtures obtained were finally molded in plastic buckets (20 × 30 mm), cooled and stored at refrigeration temperature (4 °C for 24 h) until further use. To obtain both single systems, the melted CB and the OG were directly molded and stored under identical conditions. 

### 2.4. Fatty Acids (FAs) Profile of CB:OG Systems

FA contents of control CB (100:0 system), CB:OG mixed systems (50:50, 40:60, 30:70 and 20:80 systems), pure OG (0:100 system), as well as of HORSO were determined (in triplicate) by saponification and bimethylation using C13:0 as internal standard. Fatty acid methyl ester (FAME) was analyzed by gas chromatography on an Agilent gas chromatograph (Model 7820A, CA-USA) fitted with a GC-28 Agilent DB-23 capillary column (60 m × 250 µm × 0.25 μm), and a flame ionization detector was used. Injector and detector temperatures were 250 and 260 °C, respectively. The temperature profile of the oven was as follows: initially 50 °C (held for 1 min), increasing 25 °C/min up to 175 °C and 4 °C/min up to 230 °C (held for 16 min). FAs were identified by comparing retention times with a FA standard (47015-U Supelco PUFA No.2 Animal Source, Sigma-Aldrich Co., St. Louis, MO, USA). FAs were expressed as mg of fatty acid/g sample. Three replicates were performed with samples prepared on two different days.

Based on the FAME results, the atherogenic index (AI) and thrombogenic index (TI) were computed as follows [30]:

AI = (C12:0 + 4 × C14:0 + C16:0)/[ΣMUFA + ΣPUFA (n − 6) and (n − 3)](1)

TI = (C14:0 + C16:0 + C18:0)/(0.5 × ΣMUFA + 0.5 × ΣPUFA(n − 6) + 3 × ΣPUFA(n − 3) + (n − 3 PUFA)/(n − 6 PUFA)).(2)

### 2.5. Rheological Measurements

Rheological measurements of the CB:OG systems were carried out with a rotational Kinexus pro rheometer (Malvern Instruments Ltd., UK) equipped with rSpace of software and a Peltier Plate cartridge in the lower plate for temperature control (resolution to 0.01 °C). A plate-plate measuring geometry PU40:PLS61X S3335SS, with smooth upper plate of 40 mm of diameter and serrated lower plate with 61 mm, was used (1.5-mm gap). Small amplitude oscillation sweeps (SAOS) were performed to analyze the viscoelastic properties of the different systems. All the CB:OG systems were loaded on the rheometer at 20 °C. For this purpose, the control 100:0 system was first melted by placing it in a thermostatic water bath at 75 °C, allowing it to cool down to 20 °C before loading it on the rheometer. Before measurements at 20 and 10 °C, all systems were allowed to rest on the geometry for 40 min (to stabilize and restructure the sample) using time sweeps carried out at 1 Hz with a chosen stress within the linear viscoelastic region (LVR). First, to determine the extent of the LVR, stress amplitude sweeps were carried out at 20 °C and at 1 Hz varying the stress from 20 to 2000 Pa, depending on the CB replacement percentage of the system and the measurement temperature. Then, frequency sweeps were performed at 20 °C between 10 and 0.1 Hz at chosen stress within the LVR. To observe the effect of cooling the system structure, both stress amplitude and frequency sweeps were also carried out at 10 °C. For this purpose, temperature sweeps were carried out from 20 to 10 °C at a linear cooling rate of 1 °C/min, and frequency of 1 Hz at the chosen stress within the LVR. Finally, to observe the effect of heating the system structure, temperature sweeps were performed from 10 to 60 °C at a linear heating rate of 1 °C/min, and at frequency of 1 Hz within the LVR. Previously, the samples were cooled from 20 to 10 °C and allowed to rest for 40 min at both 20 and 10 °C as indicated above. From all the different tests carried out, the values of storage modulus (*G*′), loss modulus (*G*″) and loss tangent (tan *δ* = *G*″/*G*′) were recorded. All the measurements were repeated at least three times, with samples prepared on different days (two batches) and performed 24 h after the formulation of the different CB:OG systems.

### 2.6. Texture Measurements

The evaluation of the consistency of the CB:OG systems was carried out using a TA.HDPlus Texture Analyzer (Stable Micro Systems, Ltd., Godalming, UK) provided with Texture Exponent software (version 6.1.16.0; Stable Micro Systems, Ltd., Godalming, UK) and equipped with a 30 kg load cell. A penetration test was performed using a polyoxymethylene cylindrical probe (P/10, 10 mm Ø) that penetrated the sample to a depth of 6 mm at a rate of 1 mm/s with a 0.1 N trigger force. After 24 h of storage at 4 °C, the samples were tempered and measured directly in the plastic buckets at both 10 and 20 °C. The maximum force and the area under the force-distance curve at the defined penetration distance were recorded and used as indicators for the consistency/hardness of the CB:OG systems. Three replicates were performed with samples prepared on different days (two batches).

### 2.7. Oil-Binding Capacity

Oil-binding capacity (OBC) of the CB/OG systems was measured by calculating the amount of oil expressed naturally during a defined period of time, based on the procedure described by other authors with some modifications [18,31]. A small sample disc weighing around 10 g was placed on the center of a piece of filter paper (125 mm of diameter) and subsequently stored at 4 °C for 24 h. Oil loss (OL) after 2 and 24 h at room temperature was calculated as:

(3) $$\mathrm{OL}\;\left(\%\right)=\;\frac{\left(W_{t}-W_{0}\right)}{F_{\mathrm{system}}}$$

where OL is the oil loss. W_t_ is the filter weight at time t (2 or 24 h), W_0_ is the filter weight at time 0, and F_system_ is the fat content of the formulated system. Then, OBC (%) was calculated as 100 − OL (%). Three replicates were performed with samples prepared in two different batches.

### 2.8. Statistical Analysis

For FA composition, one-way analysis of variance (ANOVA) was performed to study the CB replacement percentage effect. For viscoelastic properties and textural parameters, one-way ANOVA was performed on each property or parameter to study the CB replacement percentage effect within the same temperature (10 and 20 °C) and the temperature effect within the same CB replacement percentage (0, 50, 60, 70, 80 and 100%). Identical analyses were conducted for the OBC, but in this case the percentage of replacement of CB and the time period at room temperature (2 and 24 h) were the two main effects studied. Significant differences between pairs of means were evaluated by the Tukey test, using a 95% confidence interval (*p* < 0.05). Analyses were carried out using IBM SPSS statistical for Windows, Version 26.0 (IBM Corp., Armonk, NY, USA).

## 3. Results and Discussion

### 3.1. FA Composition of CB:OG Systems

The fatty acid (FA) composition of the genuine CB, the oil employed to develop the OG (HORSO), the pure OG, as well as the different mixed systems CB:OG, are presented in Table 1. 

Predominant FAs in the CB composition (100:0) are stearic, oleic, and palmitic acid, with linoleic acid present in a lesser proportion. This represents SFA and unsaturated fatty acid (UFA) compositions of nearly 60 and 35% respectively. This agrees with the FA composition of natural CB produced in various countries [32]. Conversely, HORSO is characterized by low SFA and PUFA contents and an elevated quantity of MUFAs (>80%), in particular oleic acid, which corresponds to 79% of its total FA constituents. As expected, the FA composition of the OG (0:100) is essentially similar to that of HORSO, which was not included in the statistical analysis as it is not a CB:OG system.

The percentage of CB substitution with OG has a significant effect (*p* < 0.05) in the FA composition of the different formulated CB:OG systems. Increasing the OG proportion in the system entails a significant reduction in the SFA content and an increase in unsaturated fats (UFAs), mostly MUFAs and to a lesser extent PUFAs, which is consistent with the composition of the ingredients of the mix. At a nutritional level, the FA composition of CB is not in line with health recommendations [4], hence, the partial substitution of CB with the OG (even in the lowest percentage of substitution) is a strategy that provides an ingredient with an improved lipid profile and, thus, could be an adequate replacement for CB in the formulation of confectionery products. 

Improvements in the lipid profile observed in the mixed systems CB:OG are associated with the reduction of SFAs, such as palmitic acid, which is associated with the appearance of certain chronic degenerative diseases (cardiovascular disease, some types of cancer, etc.). There is evidence of the relationship between high palmitic acid consumption and an increased level of total cholesterol and low-density lipoprotein (LDL) cholesterol in blood [33]. On the other hand, the improved lipid profile of the systems is linked to a significant increase in oleic acid, which has several known beneficial effects, particularly on cardiovascular health [34]. Nonetheless, it should be noted that although CB has an elevated content of stearic acid, this FA is considered to be neutral in relation to cardiovascular disease, since it neither increases blood cholesterol levels [35], nor contributes to any other thrombogenic risk factor [36].

The nutritional quality of the lipid fraction of foods can be evaluated according to the PUFA/SFA ratio. It has been pointed out that an increase in this proportion can lead to a reduction in plasma total cholesterol, and it is advisable to increase this ratio in different foods [37]. The PUFA/SFA ratio in CB was approximately 0.05 (Table 1), while the strategy of partial and total replacement of CB by the OG lead to a significant increase in the ratio, which was almost 6 times higher in samples with a greater percentage of substitution of CB with the OG (30:70 and 20:80), being closer to the recommended values (>0.4) [38]. Likewise, the atherogenic index (IA) and thrombogenic index (TI) of the systems were determined (Table 1), a significant decrease (*p* < 0.05) of both indexes occurred as the substitution of CB with OG increased, fundamentally due to the reduction in SFAs and increase in UFAs (MUFAs and PUFAs). The difference in the lipid composition of the CB:OG system formulated will affect the rheological and textural properties; a fat crystal network is based on its lipid composition and directly related to its macroscopic properties [39].

### 3.2. Linear Viscoelastic Properties of CB:OG Systems

**Figure 1:**
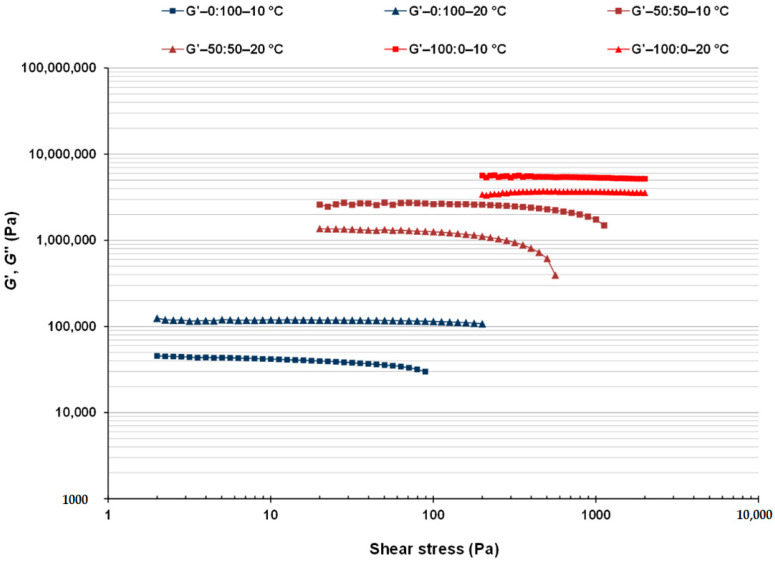
Storage modulus (*G*′) as a function of the applied shear stress at 100:0, 50:50 and 0:100 formulated systems at 20 and 10 °C.

To study the effect of the internal oil gelation by HPMC on the structural properties of the CB:OG systems, SAOS rheological measurements were carried out. Results were compared to those of the CB single system (100:0 ratio), where only CB provides structure and no OG is present. Stress amplitude sweep tests were initially run to determine the LVR of all the different CB:OG systems. The evolution of *G*′ by increasing the applied stress corresponding to systems with 50 and 100% of CB replacement (50:50 and 0:100 ratios, respectively) in comparison with the control 100:0 system at 10 °C and 20 °C is shown in (Figure 1). Mixed systems with partial CB replacement ranging between 60 and 80% (40:60, 30:70 and 20:80 ratios) exhibited decreasing intermediate *G*′ values between those of 50:50 and 0:100 formulated systems and narrower LVRs as the percentage of replacement of CB by the OG increased. In the LVR, the gel strength of a sample can be evaluated from the *G*′ value in the LVR (*G*′_LVR_) and the viscoelastic limit, i.e., the critical or maximum value of the amplitude sweep (shear stress, *σ*_max_) at which the *G*′ value shows a noticeable deviation from the previously constant values [3,40]. It is possible to clearly appreciate that both the percentage of replacement of CB and the measurement temperature had a significant effect on either the *G*′_LVR_ or the *σ*_max_ values (data values not shown). Nevertheless, the 50:50 mixed system showed a resistance to the applied stress closer to that of the 100:0 control system than to the 0:100 system. Please note that the 50:50 and 100:0 samples present *G*′_LVR_ values in the same logarithmic decade, whereas those corresponding to pure OG (0:100) are almost two decades below. 

Even though the increase in the replacement percentage of CB was accompanied by a decrease in the *G*′_LVR_ value, the presence of a higher quantity of the OG in the system also had a significant effect on the *σ*_max_ value, and in consequence, on the extension of the LVR. The lowest extent of linear viscoelastic response was found in both temperatures in the 0:100 system, this is, in the pure OG system without CB (Figure 1). In this system, *σ*_max_ values ranged between approximately 20 and 100 Pa, depending on the measurement temperature. Similar LVRs, although showing lower *G*′_LVR_ values (<10,000 Pa), were obtained by Patel et al. [17] in oleogels prepared from sunflower oil emulsion in water, stabilized with methylcellulose and xanthan gum. According to these authors, the complete extraction of the water phase by drying results in the formation of a structure in which oil drops are found tightly packed in a polysaccharide network. Also, Patel et al. [11] reported a *σ*_max_ value somewhat higher than that of 100 Pa obtained in oleogels developed by using the emulsion-templated approach and olive oil, although these authors used a mix of gelatin and xanthan gum as stabilizer as well, and observed an increase in the *σ*_max_ value with the concentration of both hydrocolloids. 

Compared with the 0:100 system, the 50:50 system shows a notable extension of the LVR with *σ*_max_ values that are close to 500 Pa at 10 °C. At the same time, in the 100:0 control system without OG, the *σ*_max_ values are above 1000 Pa for both temperatures. This result indicates that fat crystals are the dominating structure that provides a higher resistance against structure degradation for systems constituted by CB as well as by OG.

Regarding the measurement temperature, and in the presence of CB, the *Gʹ*_LVR_ values were higher at 10 than at 20 °C in all samples, although differences were more noticeable in the 50:50 system (Figure 1). This result is attributed to the more complete crystallization of cacao fat that takes place in the systems at 10 °C. In this context, Bahari and Akoh [28] provided temperatures for the onset and completion points of cacao fat crystallization, determined by differential scanning calorimetry (DSC), of 15.5 ± 0.1 °C and of 6.3 ± 1.1 °C, respectively. On the contrary, in the 0:100 single system, higher sensitivity to the applied stress was observed at 10 than at 20 °C, evidence of a lower resistance against structural degradation at the lower temperature. However, for both temperatures the phase angle of the 0:100 system in the LVR was less than 10 °C, suggesting a phase response to the sinusoidal stress applied that is characteristic of viscoelastic solids [17].

Nevertheless, and though the OG alone presents the lowest *Gʹ*_LVR_ values and a significantly narrower LVR than the rest of the systems containing CB, it must be pointed out that the structural three-dimensional network formed by HPMC provides the necessary structure to show a solid-like behavior, retaining the liquid oil in the developed OG. Meng et al. [13] applied stress sweeps to oleogels elaborated with soy oil using different combinations of HPMC with other thickeners, such as xanthan gum, carboxymethyl cellulose, guar gum, etc. as organogelators. *G*′_LVR_ values were lower than 1000 Pa and, in addition, the *σ*_max_ values were not above 1 Pa for any of the HPMC combinations with the aforementioned polysaccharides. The same authors also developed oleogels with xanthan gum (0.3%) combined with HPMC in concentrations that ranged from 0.2 to 1% [14]. Although the increase in HPMC concentration incremented gel resistance in the oleogels, the LVR extension did not exceed 1 Pa in any of the cases.

Thus, in this study, using an emulsion-templated approach with HORSO, a concentration of HPMC of 1%, and without the use of any additional thickening agent, an OG with a superior stability and a more compact network than any previous one developed by any other author, has been obtained. Yet, and from the variation of the values of storage modulus (*G*′) versus the shear stress wave amplitude (Figure 1), it is possible to infer that the obtained OG, alone, could not totally replace CB in confectionery products and confer the same structural properties.

**Figure 2:**
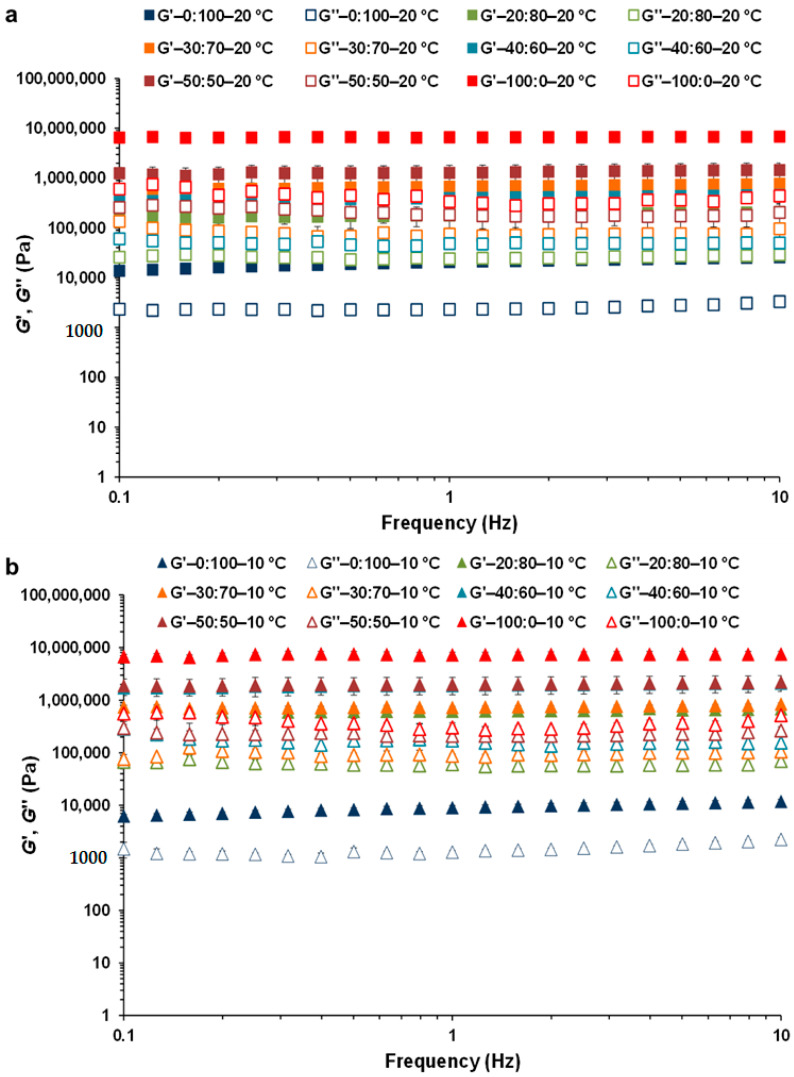
Storage modulus (*G*′: filled symbols) and loss modulus (*G*″: open symbols) as a function of oscillation frequency for the different formulated CB:OG systems: (**a**) at 20 °C; (**b**) at 10 °C.

Subsequently, mechanical spectra of the different CB:OG systems were obtained to carry out a linear viscoelastic characterization in function of the frequency. Initially, these spectra of the systems at 20 °C were obtained by applying a constant stress in the LVR (Figure 2a). However, given the importance of the temperature of crystallization and the size distribution of the fat crystals in products containing CB [24], to study the effect of cooling on the structure of the different systems, the mechanical spectra were also obtained at 10 °C (Figure 2b). 

At 20 °C, and in the 6 formulated CB:OG systems, the *G*′ value is higher than that of *G*″ in the complete interval of analyzed frequencies (Figure 2a), and with just a small frequency dependence mainly of the elastic or storage modulus *G*′, which was slightly more and less perceptible in the systems elaborated with OG and CB alone, respectively.

This low frequency dependence of both viscoelastic moduli would indicate the existence of a strong internal network in all systems [8]. In addition, *G*′ values in the 100:0 control system present a difference of almost a logarithmic decade with respect the values corresponding to the 50:50 system, which is of about three logarithmic decades if compared with the system based on OG alone (0:100). A solid-like mechanical behavior is characterized by a *G*′ which is far larger than *G*″ and both moduli are independent of frequency [41], as found in the 100:0 system. However, the 0:100 system showed a structured liquid behavior in which *G*′ is also larger than *G*″, but both moduli are slightly dependent on frequency, which is characteristic of gels where one of the components is a liquid present in a substantial quantity, as is the case of the oil retained in the OG developed. In turn, the CB:OG mixed systems exhibited an intermediate state that resembles a behavior of structured soft solid as the replacement percentage of CB by the OG increased (Figure 2a). Hence, a clear effect of the concentration of both ingredients on the values of the rheological properties of the CB:OG systems can be observed.

At 10 °C, the shape and distribution of the mechanical spectra obtained for the CB:OG systems (Figure 2b) are very similar to the corresponding ones at 20 °C, and even though the highest *G*′ and *G*″ values also corresponded to the control system elaborated without OG, there is scarcely any difference in the *G*′ values of the 50:50 and 40:60 systems, being the case that for all the systems *G*′ values are closer at 10 than at 20 °C. Conversely, the mechanical spectrum of the pure OG at 10 °C is more separated from the corresponding spectra of the mixed systems at 20 °C. 

At the same time, *G*′, *G*″ and tan δ values of the formulated CB:OG systems associated with a frequency of 1 Hz, and obtained at both temperatures, 10 and 20 °C, are gathered in (Table 2). The one-way ANOVAs indicated that the replacement percentage of CB had itself a relevant effect in the three rheological properties at 20 as well as at 10 °C. At both temperatures, and compared with the 100:0 control, *G*′ and *G*″ values dropped significantly as the percentage of substitution of CB with the OG increased. At 20 °C only the *G*′ and *G*″ values for the 30:70 system are greater than the ones for the 40:60 system, and their tan *δ* values are also lower. In turn, at 10 °C, there are essentially no significant differences among the tan δ values of the systems elaborated with a partial substitution of CB. At 20 and 10 °C the highest values for *G*′ (6630 ± 83.5 kPa and 7281 ± 848 kPa, respectively) and *G*″ (339 ± 12.7 kPa and 305 ± 69.2 kPa), as well as the lowest tan *δ* values (0.051 ± 0.001 and 0.042 ± 0.011, respectively) correspond to the genuine CB (Table 2), which reflects that the CB system, at either temperature, presents an internal network that is stronger, more cohesive, and showing higher connectivity than the rest of the systems with growing percentages of OG. Certainly, the 100:0 control system contains fat crystals that generate an internal highly ordered three-dimensional network which provides high resistance and hardness to the structure [24]. According to Bahari and Akoh [24], CB crystallizes in a polymorphic *β* structure responsible for a melting point close to the body temperature, and in morphological terms, CB presents a mix of granular crystals and small spherules that are associated with its high SFA content (Table 1). The polymorphic behavior of CB has been studied by some investigators [24,28], and, even in the presence of sunflower oil [21], it is determined by its high solid fat content (SFC) and specific composition of TAGs. Chai et al. [39] have also reported that the thickness of the nanoscale-crystals decreases significantly with a higher SFC. In contrast, the crystalline nucleus of fats presents a slower velocity of crystallization and growth in the CB:OG mixed systems that have a lower SFC. 

On the other hand, the softer structure is that of the 0:100 system, elaborated without CB, with *G*′ values of 20.8 ± 1.25 kPa and 9.04 ± 0.494 kPa and *G*″ values of 2.33 ± 0.158 kPa and 1.29 ± 0.084 kPa at 20 and 10 °C, respectively. However, in the 50:50 system, although *G*′ values are also significantly lower than those of the 100:0 control at both temperatures, the existing differences are much smaller, and even at 10 °C there are no significant variations between the *G*″ values corresponding to the 50:50 and 100:0 systems (Table 2). The results also reflect that in the systems elaborated with CB and OG, the fat crystals of CB are the dominant structure reinforcing the OG, and their rigidity increases as the OG concentration diminishes. The results, hence, corroborate that SFAs contribute by providing more solidity to fat, whereas the opposite is true in the case of fats with a high UFA content [1]. 

Regarding the measuring temperature in each formulated system, although in the 100:0 control *G*′ and tan *δ* values at 10 °C are higher and lower respectively than at 20 °C, the differences are minor (Table 2), while these same differences proved to be significant in the case of the 50:50 system. At the same time, in the 40:60, 30:70 and 20:80 mixed systems, *G*′ values as well as *G*″ values were significantly higher at 10 than at 20 °C, as was the case for systems 40:60 and 20:80, where tan δ values were also significantly lower at 10 °C. In the pure OG temperature had a significant effect on *G*′ and *G*″ values as well. Nonetheless, in absence of CB *G*′ and *G*″ were higher at 20 than at 10 °C, although the viscoelasticity of the system (tan *δ*) did not suffer any variation with temperature. In reference to this last rheological property, tan δ values are below 0.1 for both the 100:0 control system at 10 and 20 °C, and the 50:50 system at 10 °C, therefore they can be characterized as strong gels [41]. Nonetheless, it should be noted that in all systems, including 0:100 for pure OG, tan *δ* values are very low and below 0.15.

In addition, it is interesting to reiterate that the 0:100 system developed with the OG alone, exhibits *G*′ and *G*″ values greater than those of any other oleogel, found in the literature, and formulated with a cellulose ether combined with a thickening agent. For instance, oleogels elaborated with HPMC and xanthan gum showed *G*′ values at 1 Hz and at 25 °C that were below 1 kPa [14,15], while in methylcellulose and xanthan gum [17], and gelatin and xanthan gum oleogels [11], these values were below 10 kPa. Likewise, Jiang et al. [21] obtained oleogels also by an emulsion-templated approach, but from regenerated cellulose and carboxymethyl cellulose, obtaining *G*′ values close to 15 kPa. All these *G*′ values are inferior to the ones obtained for the 0:100 system at 20 °C (Table 2).

**Figure 3:**
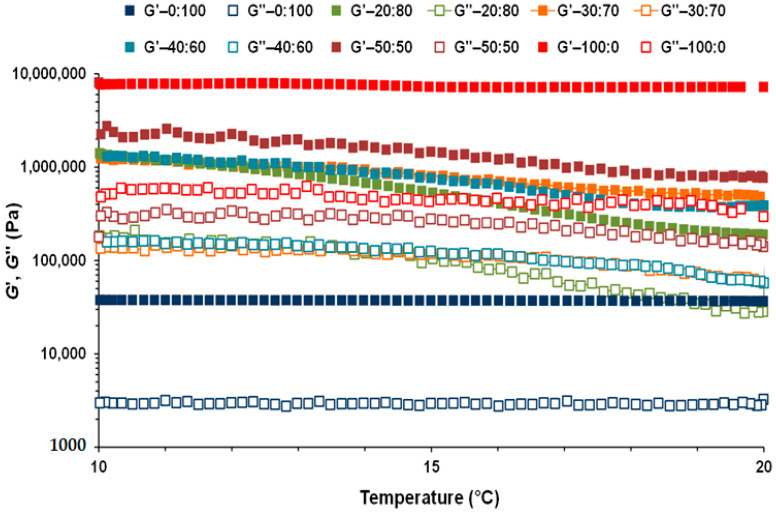
Storage modulus (*G*′: filled squares) and loss modulus (*G*″: open squares) as a function of temperature 20 and 10 °C for the different formulated CB:OG systems.

As mentioned above, to study the cooling effect in the structure of the CB:OG systems it was necessary to previously perform temperature sweeps from 20 to 10 °C. Figure 3 shows the evolution of *G*′ and *G*″ values from a temperature of 20 to 10 °C in the different CB:OG systems, while (Table S1) has *G*′, *G*″ and tan *δ* values at 20, 15 and 10 °C obtained from these coolings. It can be appreciated in (Figure 3) that *G*′ and *G*″ values of the 100:0 control and the 0:100 pure OG systems, remain almost constant between 20 to 10 °C, as opposed to the viscoelastic properties corresponding to the mixed CB:OG systems, which are much more sensitive to the temperature coolings performed, and increase significantly as the temperature goes down. This fact is associated with the hardening of the system structure because of the solidification of the cacao fat crystals, which leads to the formation of an internal three-dimensional network of crystals that is more structured and has greater consistency [8]. Bahari and Akoh [24] observed that the crystallization onset temperature for CB fat was 15.5 °C, and slightly superior (16.1–18.2 °C) for different CB equivalents synthesized by enzymatic interestification. However, and although it cannot be observed in (Figure 3), *G*′ and *G*″ values in the 100:0 system also increased during cooling to 10 °C (Table S1) due to the strengthening of its network structure linked to the temperature decrease. 

At 20, 15 and 10 °C the replacement percentage of CB had a significant effect on three viscoelastic properties values presented in (Table S1), decreasing both *G*′ and *G*″ as the replacement percentage of CB by the OG increased. Also, regarding the measuring temperature of each formulated system, in both the 100:0 and the 0:100 systems, this factor did not show a relevant effect on the values of the viscoelastic properties (Figure 3; Table S1). Conversely, in the 50:50, 40:60, 30:70 and 20:80 systems the measuring temperature had a significant effect on the *G*′, *G*″ and tan *δ* values, increasing both moduli and decreasing loss factor when lower in temperature.

**Figure 4:**
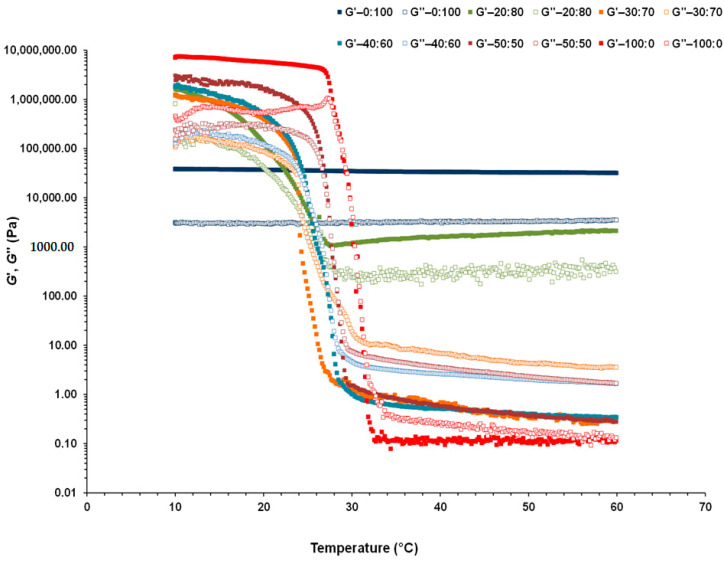
Storage modulus (*G*′: filled squares) and loss modulus (*G*″: open squares) as a function of temperature during heating between 10 and 60 °C for the different formulated CB:OG systems.

Another very important aspect that characterizes the functionality of the formulated CB:OG systems is their thermorheological behavior, since it provides important structural information for its applicability in product formulations. In fact, a typical characteristic of CB and cocoa derivatives is their melting profile. Thus, to observe the concurring changes in the structure and detect the melting points (cross-over temperature for *G*′ and *G*″) of the different CB:OG systems, temperature sweeps were also performed for dynamic thermomechanical analysis (DTMA) from 10 to 60 °C in the LVR (Figure 4). It is possible to observe how in the 0:100 system, *G*′ and *G*″ values were kept constant from 10 to 60 °C. Similar results were obtained by Tavernier et al. [3] in oleogels prepared with soy protein isolate, submitted to DTMA from 5 to 80 °C, and by Patel et al. [16] in oleogels containing HPMC and xanthan gum and subjected to heating from 10 to 70 °C. This result reflects that the network structure of the developed OG in this study is very thermostable against heating. Evidently, by drying at 60 °C, eliminating the water from the sunflower oil emulsion stabilized with HPMC creates a network structure in which the oil drops remain tightly packed, and, at the same time, this structure lasts after shearing to obtain the OG, preventing oil release and corroborating previous studies [11,18,21]. 

However, compared with the 0:100 system, in the formulations where CB is the fat source it is possible to distinguish two types of behavior as temperature is increased, as this behavior is also a function of the replacement percentage of CB by the OG in the system. Thus, the thermorheological behavior of the 20:80 system (20% CB and 80% OG) was significantly different to that of the 0:100 system without CB, as well as of the rest of the systems with lower OG percentages (Figure 4). In the 20:80 system, from 10 to 30 °C the *G*′ and *G*″ values drop abruptly, although they do not cross, and both *G*′ and *G*″ values show a slight increase from 30 to 60 °C. Therefore, in this system the elastic structure of the OG is still dominant, showing a structured soft solid-like thermomechanical behavior with *G*′ values above those of *G*″ all throughout the heating process. 

In contrast, in the systems containing a higher amount of CB, the thermorheological behavior was very different (Figure 4). In the 100:0, 50:50, 40:60 and 30:70 systems, initially, a severe decrease in both moduli can be observed in the first stages of heating, followed by a cross-over of the *G*′ and *G*″ values connected to the melting of the CB fat crystals, and finally the systems show a liquid-like behavior as demonstrated by *G*″ values higher than those of *G*′. This might be because more crystalline TAGs melted at a higher temperature and resulted in a lower SFC and less crystal aggregation [39]. In addition, a strong correlation can be observed between *G*′ and *G*″ values and the quantity of OG added in the CB:OG systems (Figure 4). As SFC decreases by increasing OG incorporation, a higher proportion of the product is solid between 30 and 60 °C.

Table 3 shows the average values of the viscoelastic moduli corresponding to 10, 20, 30 and 40 °C for the different CB:OG systems, as well as the temperature in which the cross-over between *G*′ and *G*″ values occurs in the samples, given that such cross-over takes place. The cross-over temperature of the viscoelastic moduli can be considered to be a melting point measurement obtained by DSC [31]. In all the formulated systems *G*′ and *G*″ values at 50 and 60 °C were very similar to those obtained at 40 °C (Figure 4), and consequently, they are not presented in (Table 3). At 10, 20, 30 and 40 °C the replacement percentage of CB by the OG had a significant effect in the values of both *G*′ y *G*″. As expected, at 10 and 20 °C, *G*′ and *G*″ values decrease significantly as the percentage of OG in the systems increases, On the contrary, at 30 and 40 °C the highest *G*′ values correspond to the most thermostable system, 0:100, constituted by pure OG (Table 3). Additionally, just in the pure OG system, the measuring temperature did not have a relevant effect on the viscoelastic moduli. On the other hand, the average temperature corresponding to the *G*′ and *G*″ cross-over was that of 29 °C in the 100:0 control system and, compared with the genuine CB, this temperature decreased significantly in the systems with a partial replacement of 50, 60 and 70% of CB by the OG (Table 3). These results reflect that as the SFC increases in the system by decreasing the OG proportion, the process requires more energy to melt more crystalline fat compared with the CB:OG systems with a higher OG content.

The differences in melting temperatures between the genuine CB and all the different CB:OG mixed formulations give insight into the behavior of the mixtures as a system. If there was no interaction between CB and oil droplets, CB melting temperature would be the same in the CB:OG mixed systems and the control 100:0 one. However, the melting profile of each CB:OG mixed system is shifted lower in temperature by increasing the OG content (Figure 4). This shift indicates an interaction between the oil droplets and the CB, producing the softening effect observed. It would appear that the slight stirring applied during the preparation of the CB:OG mixed systems supplies enough energy to produce fat globules within the oily phase provided by the OG, and possibly emulsified by HPMC. Similar results were reported by McGill and Hartel [42] from melting profiles using DSC for chocolate and ganache formulations made with different ratios of milk fat procured from cream and butter. The cited authors also found that in both ganache and chocolate, melting enthalpies decreased with decreased SFC. 

Bahari and Akoh [24] obtained by DSC onset and completion melting temperatures of the fat crystals in CB at 30.9 and 38.6 °C, respectively. As well, onset and completion melting temperatures also obtained by DSC in chocolate were 29.6 and 38.6 °C, respectively [42]. Hence, the 29 °C corresponding to the 100:0 system cross-over, are quite proximal to the melting temperature for CB documented by other authors. 

### 3.3. Texture Measurements of CB:OG Systems 

**Figure 5:**
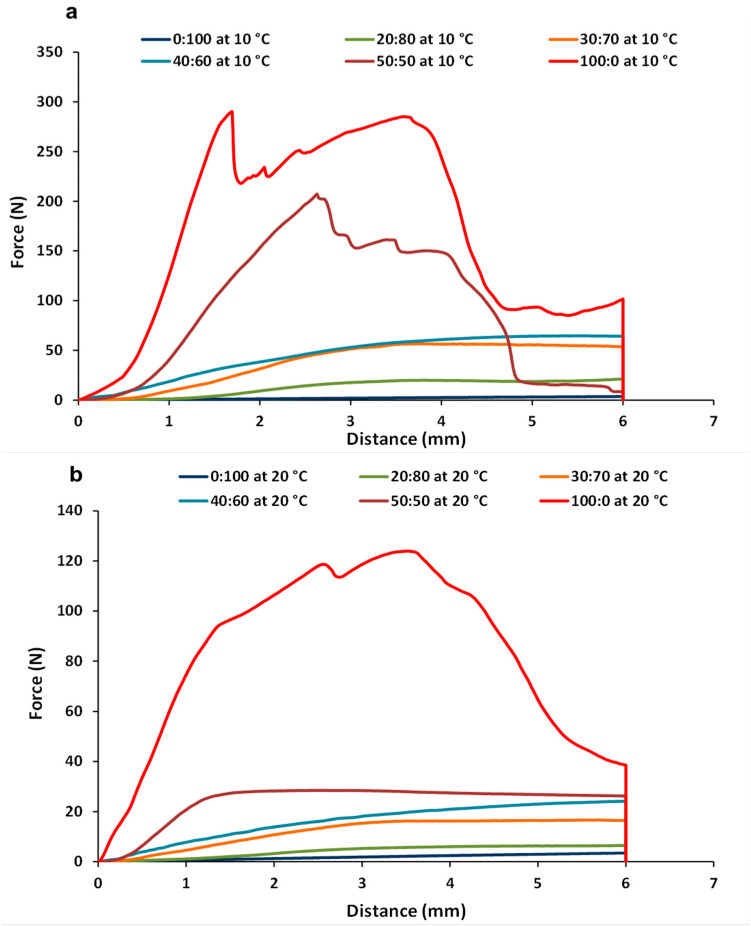
Force-distance curves of the different formulated CB:OG systems measured: (**a**) at 10 °C; (**b**) at 20 °C.

To understand the resistance that the CB:OG systems present against large deformations, their mechanical properties were determined at 10 and 20 °C by a penetration test. Figure 5 shows the texture profiles (force-distance curves) corresponding to the different CB:OG systems formulated. At both temperatures, the 0:100, 20:80, 30:70 and 40:60 systems with a higher percentage of OG presented force/distance profiles distinctive of a compact but malleable gel structure, in which force increases with the distance travelled by the probe, and peaks indicating the breakdown of the structure are not observed, nor does a final drop of the force increase with distance. However, at 10 °C, both the 100:0 control and the 50:50 mixed systems with a higher content of cocoa solids (Figure 5a) showed very different profiles with a first force peak that was reached almost immediately after sample penetration, which reflects an initial rupture of the structure that was concurrent with the characteristic “snap” of chocolate and by a final force drop. This last sound, together with high hardness, is a desirable attribute in chocolate [28]. In the 100:0 samples at 20 °C (Figure 5b), one or various peaks related to structural rupturing during probe penetration were detected, and they, as well, were concurrent with the aforementioned “snaps”, although not in such a distinctive manner as observed at 10 °C. At 20 °C the structure of the 100:0 control system is not as brittle as at 10 °C, since some TAGs start melting at this point and, consequently, less work is required to deform the system at a higher temperature [6]. 

The textural parameter derived from the curves force-distance corresponding to the CB:OG formulated systems and determined at 10 and 20 °C are presented in (Table 4). At both temperatures, the replacement percentage of CB by the OG has a significant effect on both textural parameter values, which diminish progressively as the OG percentage increases in the system. Again, the differences in SFC of the mixed systems would explain why formulations with more CB (more SFC) were harder than those with more OG. Both the oily and dispersed (globules of fat) phases influence textural properties of the CB:OG mixed systems. Higher levels of crystalline fat in the dispersed phase resulted in firmer systems, as previously observed from the textural properties of ganache and caramel [42,43]. A stronger hardness has been also associated with higher SFC and a denser aggregation in fat crystal networks [39].

In relation to the temperature effect, the presence of CB in the systems, even at a low percentage (20%), led to higher values in maximum force and area under the force-distance curve (AUC) at 10 than at 20 °C, corroborating the results derived from the rheological tests. Additionally, this result also reflects that at 10 °C cacao fat crystallization is almost complete, which means an increment of the system consistency and a hardening of the structure [24].

Conversely, in the 0:100 system, the maximum force and the AUC were significantly higher at 20 than at 10 °C, which reflects a greater gel-like consistency in the system formulated with the OG alone at a higher temperature. This result is also in consonance with the result obtained from the frequency sweeps at both temperatures (Table 2). The values of these textural parameters in the 0:100 system at 20 °C are similar to the ones provided previously by other authors in an OG developed under similar conditions [18], and these values increased with HPMC concentration. In other oleogels prepared by using the emulsion-templated approach, an increase in system consistency was also observed as the hydrocolloid concentration became higher [11,15].

### 3.4. Oil-Binding Capacity (OBC) of CB:OG Systems 

OBC is an important property in a lipid system, and indicates the system capacity to retain oil in its structure, thus denoting its physical stability. The OBC values in the different CB:OG formulated systems are represented in (Table 5). The results show that either after 2 or 24 h, the effect of the percentage of replacement of CB by the OG has a significant effect in the OBC of the samples, in such way that the values decrease as the quantity of OG present in the CB:OG systems increases. Therefore, after both 2 and 24 h, neither the 100:0 control system, nor the 50:50 system, suffered any oil loss, being their OBC was that of a 100% and reflecting that in the 50:50 mixed system the oil from the OG remains totally retained and immobilized in the complex mix of structures formed. After 2 h there were no significant differences in oil loss values among the 40:60, 30:70 and 20:80 mixed systems, as these differences were very small between 0.6 and 1%. After 24 h, oil loss in the 40:60 and 20:80 systems increased significantly, and reached values up to 5.4% in the system with the greatest OG content.

Finally, although the pure OG system (0:100) had the greatest oil loss at 2 and 24 h its OBC was still very high, with values of 98.4 and 93.4% respectively (Table 5). This result shows that when 1% HPMC is added to the oil-in-water emulsion, it favors the formation of a strong physical network with a very high capacity to retain oil drops. It should be noted that the time (2 h vs. 24 h) did not have a significant effect on the 100:0, 50:50 and 30:70 systems. 

In oleogels developed with HPMC under similar conditions to the ones employed in this study, Espert et al. [18] obtained an OBC of 92.8% after 24 h. The authors just cited observed that the OBC decreased significantly to 66.2% when the HPMC concentration decreased in the emulsion from 1 to 0.5%, concluding that a minimum HPMC concentration of 1% is required to trap the oil in the OG after drying. Also, Meng et al. [14] previously provided similar results in oleogels elaborated with the same cellulose ether. In addition, these authors also observed that as the HPMC concentration in the OG increased, its OBC also increased. 

## 4. Conclusions

The use of HPMC is an adequate strategy to structure a large amount of healthy liquid oil and develop an OG with an improved lipid profile to be used as CB fat replacer. Also, this OG exhibited distinctive characteristics of a plastic and solid-like fat, which represents an essential aspect for its use as a substitute for CB, as well as for other saturated fats. Among the CB:OG formulated systems, the 50% replacement of CB by the OG is a promising technological option in the formulation of chocolates and derivatives, since it provides a healthier lipid profile and physical characteristics similar to genuine CB, as its elevated oil retention capacity, rheological, and textural properties reveal. More structural research and sensory evaluation is needed aimed at incorporating the HPMC-based OG obtained by using the emulsion-templated approach into chocolate formulations to validate the main findings of this research. 

## Figures and Tables

**Table 1:** Fatty acids (FAs) profile in the different formulated CB:OG systems and in high oleic refined sunflower oil (HORSO).

mg Fatty Acid/g Sample	100:0	50:50	40:60	30:70	20:80	0:100	HORSO
Palmitic C16:0	238.83 ± 2.05 ^A^	143.43 ± 0.069 ^B^	119.86 ± 0.78 ^C^	95.59 ± 0.53 ^D^	100.37 ± 0.58 ^E^	39.89 ± 0.067 ^F^	40.44 ± 0.10
Stearic C18:0	340.50 ± 3.34 ^A^	190.89 ± 0.19 ^B^	154.88 ± 1.33 ^C^	116.96 ± 0.94 ^D^	124.41 ± 1.00 ^E^	30.02 ± 0.015 ^F^	30.28 ± 0.074
Arachidic C20:0	10.47 ± 0.14 ^A^	6.70 ± 0.046 ^B^	5.80 ± 0.048 ^C^	4.87 ± 0.029 ^D^	5.06 ± 0.033 ^E^	2.69 ± 0.014 ^F^	2.66 ± 0.019
Behenic C22:0	1.71 ± 0.040 ^F^	5.85 ± 0.048 ^E^	6.52 ± 0.0011 ^D^	7.48 ± 0.0094 ^B^	7.29 ± 0.0077 ^C^	9.73 ± 0.029 ^A^	9.92 ± 0.029
Other SFAs	4.24	4.30	4.16	4.15	4.15	3.83	3.80
∑ SFAs	595.74 ^A^	351.18 ^B^	290.17 ^C^	229.05 ^D^	241.27 ^E^	86.45 ^F^	87.11
Palmitoleic C16:1n7	2.17 ± 0.016 ^A^	1.71 ± 0.014 ^B^	1.55 ± 0.0041 ^C^	1.43 ± 0.0018 ^D^	1.45 ± 0.0022 ^D^	1.14 ± 0.0037 ^E^	1.16 ± 0.024
Vaccenic C18:1n7	3.32 ± 0.070 ^E^	5.39 ± 0.0029 ^D^	5.76 ± 0.072 ^C^	6.25 ± 0.072 ^B^	6.15 ± 0.072 ^B^	7.39 ± 0.073 ^A^	7.77 ± 0.0039
Oleic C18:1n9	313.34 ± 2.94 ^F^	556.03 ± 0.037 ^E^	591.81 ± 0.91 ^D^	647.51 ± 0.51 ^B^	636.37 ± 0.59 ^C^	777.46 ± 0.43 ^A^	789.42 ± 2.15
Eicosenoic C20:1n9	0.640 ± 0.0086 ^E^	1.60 ± 0.033 ^D^	1.76 ± 0.037 ^C^	1.99 ± 0.046 ^B^	1.95 ± 0.044 ^B^	2.52 ± 0.068 ^A^	2.73 ± 0.083
∑ MUFAs	319.47 ^F^	564.73 ^E^	600.89 ^D^	657.18 ^B^	645.92 ^C^	788.51 ^A^	801.08
Linoleic C18:2n6	28.26 ± 0.22 ^F^	62.4 ± 0.14 ^E^	67.92 ± 0.060 ^D^	75.85 ± 0.12 ^B^	74.26 ± 0.11 ^C^	94.35 ± 0.25 ^A^	97.30 ± 0.36
Linolenic C18:3n3	1.73 ± 0.054 ^A^	1.12 ± 0.040 ^B^	1.04 ± 0.018 ^C^	0.90 ± 0.012 ^D^	0.92 ± 0.013 ^D^	0.57 ± 0.0048 ^E^	0.58 ± 0.032
Other PUFAs	-	0.30	0.23	0.28	0.27	0.39	0.38
∑ PUFAs	29.99 ^F^	63.76 ^E^	69.18 ^D^	77.02 ^B^	75.5 ^C^	95.35 ^A^	98.24
Nutritionally significant factors							
PUFAs/SFAs	0.050 ^F^	0.18 ^E^	0.24 ^D^	0.34 ^B^	0.31 ^C^	1.10 ^A^	1.12
AI	0.70 ± 0.00028 ^A^	0.23 ± 0.000073 ^B^	0.18 ± 0.00093 ^C^	0.13 ± 0.00064 ^E^	0.14 ± 0.00070 ^D^	0.047 ± 0.000071 ^F^	-
TI	3.24 ± 0.0040 ^A^	1.06 ± 0.0011 ^B^	0.81 ± 0.0053 ^C^	0.58 ± 0.0037 ^E^	0.62 ± 0.000 ^D^	0.16 ± 0.000085 ^F^	-

Values are given as mean (n = 6) ± standard deviation. ^A–F^ Effect of CB replacement percentage. For each fatty acid, mean values without the same letter are significantly different (*p <* 0.05). SFAs, saturated fatty acids; MUFAs, monounsaturated fatty acids; PUFAs, polyunsaturated fatty acids; AI, atherogenic index; TI, thrombogenic index.

**Table 2:** Viscoelastic properties at 1 Hz and at 20 and 10 °C derived from frequency sweeps for the different formulated CB:OG systems.

CB:OG	20 °C			10 °C		
	*G*′ (kPa)	*G*″ (kPa)	Tan *δ*	*G*′ (kPa)	*G*″ (kPa)	Tan *δ*
100:0	6630 ± 84 ^Aa^	339 ± 13 ^Aa^	0.051 ± 0.0013 ^Da^	7281 ± 848 ^Aa^	305 ± 69 ^Aa^	0.042 ± 0.011 ^Da^
50:50	1291 ± 20 ^Bb^	184 ± 16 ^Ba^	0.11 ± 0.0039 ^Ca^	1999 ± 25 ^Ba^	210 ± 13 ^Aa^	0.090 ± 0.015 ^Cb^
40:60	399 ± 35 ^Db^	48.5 ± 8.9 ^Db^	0.12 ± 0.012 ^B, Ca^	1900 ± 61 ^Ba^	172 ± 26 ^A, Ba^	0.11 ± 0.0079 ^B, Cb^
30:70	677 ± 2.3 ^Cb^	75.2 ± 2.6 ^Cb^	0.11 ± 0.0029 ^Ca^	732 ± 32 ^Ca^	87.9 ± 7.14 ^B, Ca^	0.12 ± 0.0048 ^A, Ba^
20:80	178 ± 15 ^Eb^	24.0 ± 3.0 ^D, Eb^	0.14 ± 0.0048 ^A, Ba^	619 ± 27 ^Ca^	60.9 ± 1.59 ^C, Da^	0.098 ± 0.0021 ^B, Cb^
0:100	20.8 ± 1.3 ^Fa^	2.33 ± 0.16 ^Ea^	0.14 ± 0.010 ^Aa^	9.04 ± 0.49 ^Cb^	1.29 ± 0.084 ^Db^	0.14 ± 0.0019 ^Aa^

Values are given as mean (n = 6) ± standard deviation. ^A–F^ Effect of CB replacement percentage. For each rheological property and the same temperature (20 and 10 °C), mean values without the same letter are significantly different (*p <* 0.05). ^a, b^ Effect of measurement temperature. For each rheological property and the same CB replacement percentage (0, 50, 60, 70, 80 and 100%), mean values without the same letter are significantly different (*p <* 0.05). *G*′, storage modulus; *G*″, loss modulus; tan *δ*, loss tangent (=*G*″/*G*′).

**Table 3:** Viscoelastic properties derived from dynamic thermomechanical analysis (DTMA) carried out between 10 and 60 °C for the different formulated CB:OG systems.

CB:OG	10 °C		20 °C		30 °C		40 °C		Temperature Cross-Over (°C)
	*G*′ (kPa)	*G*″ (kPa)	*G*′ (kPa)	*G*″ (kPa)	*G*′ (kPa)	*G*″ (kPa)	*G*′ (kPa)	*G*″ (kPa)	
100:0	7245 ^Aa^ (143)	459 ^Aa^ (122)	5764 ^Ab^ (182)	547 ^Aa^ (62)	3.29 ^Bc^ (1.2)	7.06 ^Ab^ (0.53)	0.00012 ^Bc^ (0.000095)	0.00027 ^Cb^ (0.0000074)	29.0 ^A^ (0.10)
50:50	3276 ^Ba^ (269)	277 ^Ba^ (42)	1405 ^Bb^ (121)	272 ^Ba^ (20)	0.00066 ^Bc^ (0.00014)	0.0038 ^Cb^ (0.00052)	0.00027 ^Bc^ (0.00011)	0.0018 ^Cb^ (0.00029)	27.3 ^B^ (0.47)
40:60	2096 ^Ca^ (205)	154 ^B, Ca^ (10)	653 ^Cb^ (136)	138 ^Ca^ (22)	0.0016 ^Bc^ (0.00096)	0.0072 ^Cb^ (0.0031)	0.00035 ^Bc^ (0.000092)	0.0022 ^Cb^ (0.00038)	24.3 ^D^ (0.61)
30:70	1803 ^Ca^ (109)	129 ^C, Da^ (2.7)	521 ^Cb^ (30)	118 ^C, Da^ (11)	0.00089 ^Bc^ (0.000054)	0.062 ^Cb^ (0.0066)	0.0014 ^Bc^ (0.00034)	0.034 ^Cb^ (0.0030)	25.5 ^C^ (0.31)
20:80	1605 ^Ca^ (248)	236 ^B, Ca^ (23)	127 ^Db^ (16)	40.3 ^D, Eb^ (9.3)	1.18 ^Bb^ (0.0075)	0.24 ^Cc^ (0.13)	1.62 ^Bb^ (0.080)	0.28 ^Bc^ (0.019)	-
0:100	38.1 ^Da^(1.8)	3.04 ^Da, b^ (0.16)	36.9 ^Da^ (3.5)	2.94 ^Eb^ (0.097)	34.6 ^Aa^ (3.8)	3.18 ^Ba, b^(0.13)	33.4 ^Aa^ (4.03)	3.34 ^Aa^ (0.14)	-

Values are given as mean (n = 6); values in parentheses are standard deviations. ^A–E^ Effect of CB replacement percentage. For each rheological property and the same temperature (10, 20, 30 and 40 °C), mean values without the same letter are significantly different (*p <* 0.05). ^a, b^ Effect of measurement temperature. For each rheological property and the same CB replacement percentage (0, 50, 60, 70, 80 and 100%), mean values without the same letter are significantly different (*p <* 0.05). *G*′, storage modulus; *G*″, loss modulus; tan *δ*, loss tangent (=*G*″/*G*′).

**Table 4:** Textural penetration parameters obtained at 10 and 20 °C for the different formulated CB:OG systems.

CB:OG	Maximum Force (N)	AUC (N × mm)	Maximum Force (N)	AUC (N × mm)
	at 10 °C	at 10 °C	at 20 °C	at 20 °C
100:0	229 (18) ^Aa^	408 (14) ^Aa^	137 (1.4) ^Ab^	302 (15) ^Ab^
50:50	73.5 (8.5) ^Ba^	256 (36) ^Ba^	29.2 (0.89) ^Bb^	145 (2.4) ^Bb^
40:60	64.1 (0.96) ^Ba^	248 (16) ^Ba^	20.9 (2.4) ^Cb^	80.9 (13) ^Cb^
30:70	56.1 (0.46) ^Ba^	217 (9.5) ^Ba^	16.3 (0.39) ^Db^	72.8 (4.7) ^Cb^
20:80	22.1 (1.6) ^Ca^	83.0 (5.4) ^Ca^	5.01 (0.29) ^Eb^	17.8 (1.2) ^Db^
0:100	1.38 (0.016) ^Cb^	4.47 (0.095) ^Db^	3.49 (0.24) ^Ea^	10.6 (0.84) ^Da^

Values are given as mean (n = 6); values in parentheses are standard deviations. ^A–E^ Effect of CB replacement percentage. For each textural parameter and the same temperature (10 and 20 °C), mean values without the same letter are significantly different (*p* < 0.05). ^a, b^ Effect of temperature. For each textural parameter and the same CB replacement percentage (0, 50, 60, 70, 80 and 100%), mean values without the same letter are significantly different (*p* < 0.05). AUC: area under the force-distance curve.

**Table 5:** Oil-binding capacity (OBC) for the different formulated CB:OG systems.

CB:OG	OBC (%) after 2 h	OBC (%) after 24 h
100:0	100 ± 0.000 ^Aa^	100 ± 0.000 ^Aa^
50:50	100 ± 0.015 ^Aa^	100 ± 0.01 ^Aa^
40:60	99.2 ± 0.35 ^A, Ba^	98.6 ± 0.20 ^Bb^
30:70	99.4 ± 0.61 ^Ba^	98.5 ± 0.78 ^Ba^
20:80	99.0 ± 0.19 ^B, Ca^	94.6 ± 0.35 ^Cb^
0:100	98.4 ± 0.048 ^Ca^	93.4 ± 0.28 ^Db^

Mean value (n = 6) ± standard deviation. ^A–D^ Effect of CB replacement percentage. For the same time period (2 and 24 h), mean values without the same letter are significantly different (*p* < 0.05). ^a, b^ Effect of time period at room temperature. For the same CB replacement percentage (0, 50, 60, 70, 80 and 100%), mean values without the same letter are significantly different (*p* < 0.05).

## Data Availability

Data is contained within the article or Supplementary Materials.

## Supplementary Materials

The following are available online at https://www.mdpi.com/article/10.3390/foods10040793/s1, Table S1: Viscoelastic properties derived from temperature sweeps carried out between 20 and 10 °C for the different CB:OG systems formulated.
