# Supplementary material for: Thermorheological Characterization of Healthier Reduced-Fat Cocoa Butter Formulated by Substitution with a Hydroxypropyl Methylcellulose (HPMC)-Based Oleogel
Source: Foods. 2021 Apr 7;10(4):793. doi: 10.3390/foods10040793 (PMC8067814; doi:10.3390/foods10040793)
Supplement: Supplementary file 1 [file foods-10-00793-s001.pdf]

**Table S1.** Viscoelastic properties derived from temperature sweeps carried out between 20 and 10 °C for the different formulated CB:OG systems.

| CB:OG | 20 °C | | | 15 °C | | | 10 °C | | |
|---|---|---|---|---|---|---|---|---|---|
| | *G*' (kPa) | *G*'' (kPa) | tan $\delta$ | *G*' (kPa) | *G*'' (kPa) | tan $\delta$ | *G*' (kPa) | *G*'' (kPa) | tan $\delta$ |
| 100:0 | 6207 ± 1067$^{Aa}$ | 754 ± 37.5$^{Aa}$ | 0.124 ± 0.015$^{A,B}$ | 6527 ± 178$^{Aa}$ | 592 ± 103$^{Aa}$ | 0.091 ± 0.013$^{C}$ | 7419 ± 211$^{Aa}$ | 793 ± 192$^{Aa}$ | 0.106 ± 0.023$^{A,B}$ |
| 50:50 | 1664 ± 6.50$^{Bc}$ | 172 ± 20.0$^{Bb}$ | 0.103 ± 0.012$^{B,Ca,b}$ | 2186 ± 47.0$^{Bb}$ | 280 ± 12.6$^{Ba}$ | 0.128 ± 0.009$^{Ba}$ | 3290 ± 297$^{Ba}$ | 330 ± 536.2$^{Ba}$ | 0.099 ± 0.005$^{A,Bb}$ |
| 40:60 | 638 ± 143$^{B,Cb}$ | 78.3 ± 5.39$^{C,Db}$ | 0.127 ± 0.020$^{A,Ba,b}$ | 1180 ± 330$^{Ca,b}$ | 167 ± 41.2$^{B,Ca}$ | 0.143 ± 0.005$^{Ba}$ | 1885 ± 464$^{Ca}$ | 182 ± 36.5$^{B,Ca}$ | 0.098 ± 0.005$^{A,Bb}$ |
| 30:70 | 645 ± 23.5$^{B,Cc}$ | 78.6 ± 12.4$^{Cb}$ | 0.122 ± 0.015$^{A,Ba,b}$ | 1010 ± 15.7$^{Cb}$ | 143 ± 9.45$^{Ca}$ | 0.141 ± 0.007$^{Ba}$ | 1521 ± 72.5$^{Ca}$ | 161 ± 17.0$^{B,Ca}$ | 0.106 ± 0.006$^{A,Bb}$ |
| 20:80 | 189 ± 11.4$^{Cc}$ | 28.5 ± 1.68$^{D,Ec}$ | 0.150 ± 0.000$^{Ab}$ | 533 ± 4.95$^{Db}$ | 105 ± 1.80$^{C,Db}$ | 0.197 ± 0.005$^{Aa}$ | 1391 ± 14.0$^{Ca}$ | 176 ± 6.90$^{B,Ca}$ | 0.127 ± 0.004$^{Ac}$ |
| 0:100 | 36.8 ± 0.840$^{Ca}$ | 2.85 ± 0.257$^{Ea}$ | 0.078 ± 0.009$^{Ca}$ | 37.4 ± 0.475$^{Ea}$ | 2.96 ± 0.060$^{Da}$ | 0.079 ± 0.003$^{Ca}$ | 37.8 ± 0.195$^{Da}$ | 2.99 ± 0.051$^{Ca}$ | 0.079 ± 0.002$^{Ba}$ |

Values are given as mean ($n$ = 6) ± standard deviation. $^{A\text{-}E}$ Effect of CB replacement percentage. For each rheological property and the same temperature (10, 15 and 20 °C), mean values without the same letter are significantly different ($p < 0.05$). $^{a,b}$ Effect of measurement temperature. For each rheological property and the same CB replacement percentage (50, 60, 70, 80 and 100%), mean values without the same letter are significantly different ($p < 0.05$).*G*', storage modulus; *G*'', loss modulus; tan $\delta$, loss tangent (= $G''/G'$).
